# Some Interesting Autopsies

**Published:** 1883-09

**Authors:** Frederick Peterson


### Some Interesting Autopsies.

By Frederick Peterson, M. D.

I select from sixty odd autopsies, made this year, a few which I am sure will be of interest to the profession, several from their rarety and several from their real value. For convenience I number them as they appear in my record. The specimens are all preserved in the college museum.

NO. 6—RUPTURE OF LIVER. DEATH THREE DAYS AFTER.

General Hospital—Male, æt. 35; body well nourished; in left side of frontal bone a wound one cm. in diameter penetrating to brain; anchylosis of left elbow joint at right angles, from old injury; over back of joint a scar.

Brain not examined.

*Chest:* On opening, found diaphragm on right side reaching to space between third and fourth ribs, liver close to it and invisible under ribs; transverse colon also far up under costal cartilages and xyphoid appendix; stomach pushed up; left lung in a state of engorgement, with here and there fibrin exudate in lobules; in both pleural cavities a small quantity of bloody serum; upper lobe of right lung normal, but middle and lower completely atalectatic by pressure from below; heart normal; chronic atheromatous disease of arteries generally, aorta, throughout, and bifurcations; simple fracture of eighth and ninth ribs on right side.

*Abdomen:* In peritoneal cavity, one and one-half litres of blood; spleen normal; liver exceedingly fatty, and, besides displacement upwards, a large radiating rupture near suspensory ligament, on convexity, deep into liver substance and entirely through the capsule; on under surface, near gall-bladder, an old cicatrix, possibly from an old tumor cavernosus or echinococcus cyst; stomach and intestines normal; bladder normal; in left kidney several small cysts, otherwise both normal.

*Remarks:* This man was thrown violently from a vehicle by a runaway team, and lived three days after the perforation of his skull and rupture of the liver, with internal hemorrhage. The fatty degeneration, so marked, was naturally subsequent to and a result of the rupture. Still this is not so astonishing when we consider that the liver has been known to degenerate to an equally marked degree in phosphorus poisoning in twenty-four hours.

### NO. 13—PURPURA HEMORRHAGICA.

Patient of Dr. C. Diehl, Jan. 10—May P., æt. 6 years, English; dead thirty-six hours. Body well nourished; death marks not very distinct; upon left forearm an irregular red patch, an ecchymosis, not elevated, one inch in diameter; upon right thigh several such; upon chest a large number, some fully an inch, some but the size of a pea, in diameter; there was separation of the epidermis upon the larger of these.

*Head:* Membranes and sinuses of brain normal; ventricles normal; brain substance everywhere apparently normal.

*Chest:* Lungs normal; heart normal, a few clots of pure fibrin in both sides; pericardium and endocardium normal.

*Abdomen:* Spleen slightly enlarged, not soft, and had a very peculiar appearance, as if every malpighian body were a soft white tubercle; upon examining these under the microscope they were found to consist of white round cells, some dumb-bell shape, some with protuberances, and in fact in all stages of fission. Liver was enlarged, in parts fatty, hard and with acini exceedingly well defined, cut hard (child had been taking spirit daily for a long time previous); under microscope the connective tissue of the liver was full of young cells, and the white cells were also in great numbers between the liver cells, which were fatty. Each suprarenal capsule was very large, injected, and both had large extravasations of blood in their substance; kidneys and ureters normal; Peyer's plaques in ileum swollen and with capillary injection; vermiform appendix normal, but eight inches in length; mesenteric glands much enlarged and soft; upon whole of peritoneum, both parietal and visceral, minute extravasations of blood two lines in diameter; the same were found in the mucous membranes of bladder and uterus.

*Remarks:* An autopsy on such a case is rare. Some of the lesions here noted have not been reported before. The child was, save an anæmic look, in good health until January 7th, on the afternoon of which day she became delirious. The spots appeared that day. There was no fever, but pulse frequent. Died the afternoon of next day, having had a few convulsions. This is not the place to discuss the etiology of purpura and allied diseases, and I leave it for another occasion.

### NO. 14—PUERPERAL SEPTICÆMIA.

General Hospital, January 11—Inga C., æt. 28, Norwegian emigrant; body well nourished; abdomen greatly distended.

*Head:* Brain and its membranes normal.

*Chest:* Unusual quantity of serum in pericardium; minute extravasations of blood on pericardium; soft fibrinous clots in right heart; dark, perfectly fluid blood in left heart; mitral valve thickened, grown together so as to cause stenosis, and had minute *vegetations globuleuses* upon its borders; tricuspid also thickened and with small vegetations upon its edges; fatty degeneration of heart muscle; pulmonary valve normal; aortic thickened and retraction of one lunula caused insufficience. A little serum in both pleural cavities; both pleuræ free from adhesions, but contained numerous ecchymoses, and on right side a little lymph; right lung was œdematous and contained four hemorrhagic infarcts; glands in mediastinum much enlarged, one containing five or six tubercles; left lung contained six infarcts from two to five cm. in diameter.

*Abdomen:* Stomach enormously distended, reaching to um bilicus, filled with brown, grumous fluid, its capillaries intensely congested, some bleeding. Acute inflammation of spleen and five infarcts in that organ; liver enlarged and fatty; walls of gall-bladder thickened with catarrh; pyelitis in both kidneys, in left kidney five infarcts, in right kidney two infarcts; in peritoneal cavity, one litre of serum containing fibrin; ovaries and vessels of broad ligaments all normal; vessels of mucous membrane of uterus much engorged and membrane roughened; at fundus, the inflammation of mucosa almost diphtheritic in character.

*Diagnosis:* Puerperal septicæmia.

*Remarks:* This woman had been delivered fifteen days before her death. It is noteworthy to see how general was the disease. There was a slight peritonitis, and a little pleuritis; there was endocarditis as a result of blood infection, and this was followed by the formation of renal, splenic and pulmonary infarcts. As in all acute specific fevers, there was fatty metamorphosis of liver, heart and kidneys, and softening of spleen. The stenosis of mitral, and insufficience of aortic valves were lesions, of course, of long standing. The point of infection in this case was evidently the uterine mucosa, which was found inflamed.

NO. 16—URETHRAL STRICTURE. PYEMIA.

General Hospital, Jan. 12—McMillan, æt. 42, English; dead twelve hours; body emaciated; rigor mortis marked.

Brain not examined.

*Chest:* Old adhesions at apex of left lung, with six or seven small tubercles; same in right lung, with adhesions at base; in middle lobe of right lung, anterior surface, perforation from a cavity containing green fetid pus; two other such pus depots in other parts of the same lung near surface; marks of old pericarditis; mitral valve thickened; otherwise heart normal.

*Abdomen:* Spleen enlarged and very soft. Liver slightly enlarged, soft and fatty. Both kidneys enlarged, soft and with roughened surface; pyelitis; in one kidney two old infarcts. Bladder contracted; three diverticula; mucous membrane intensely congested, and in lower part several diphtheritic patches. The urethra presented the following: one large stricture in middle of spongy portion, with false passages filled with pus; strictures in membranous portion, with two false passages, one leading to a scrotal abscess. The prostate gland was hypertrophied. There was catarrh of the rectum. All other organs normal.

*Diagnosis:* Pyemia from stricture and false passages leading to suppuration, diphtheritic cystitis, pyelonephritis and metastatic abscesses in right lung.

*Remarks:* It is but proper for me to state that these false passages in the urethra were made before entry of patient into the hospital.

NO. 48—URETHRAL STRICTURE. PYELONEPHRITIS.

General Hospital, June 4—Wm. F., æt. 35; dead twenty-eight hours; rigor mortis still present; body well built and slender; small sores about nose and mouth; scrotum about five times normal size, and, with penis, black with gangrenous blisters and putrid in odor.

Head not examined.

*Chest:* Hypostatic congestion and œdema of lungs; no adhesions of pleural surfaces. Old adhesions of apex of heart to pericardium in front; beginning atheroma of aorta; heart walls somewhat fatty; valves normal.

*Abdomen:* Spleen double usual size, very soft and flabby. Liver fatty. Both kidneys enlarged, with dilated pelves and pyelitis, and very soft and flabby. Bladder full of bloody urine, and mucosa covered with ecchymoses. Urethra full of strictures and false passages leading into scrotum below, where were numerous abscesses and blood clots. Ileum normal. Some gastritis.

*Diagnosis:* 1. Stricture of urethra, with false passages. 2. Extravasation and infiltration of urine into cellular tissues. 3. Gangrene of scrotum, cystitis and pyelonephritis as a result.

NO. 20—AIR IN THE VEINS.

Erie Co. Almshouse, Feb. 1—L. M. V., female, æt. 18, prostitute; body well nourished; medium size.

*Head:* No change in brain or meninges.

*Chest:* Thyroid gland enlarged. Blood-tinged mucus in trachea. Two ounces of sanguinous serum in pericardium; hundreds of minute extravasations on pericardium; in *left* heart, usual fibrinous clot, with *black* fluid blood; in *right* heart, a small amount of *bright red* foamy blood and no clot; musculature of heart somewhat fatty. Old adhesions on right side of chest; none on left; purulent bronchitis; great hypostatic congestion of lungs; two hemorrhagic infarcts in right lung, lowest lobe, nearly size of walnut.

*Abdomen:* Minute extravasations of blood all over omental peritoneum. Spleen normal. Liver in superficial parts fatty in patches; left lobe very large, in close connection with spleen and completely covering stomach. Congestion of suprarenal capsules. Great congestion of kidneys, and marked fatty degeneration of cortex. Ends of fallopian tubes engorged with blood. Bladder greatly distended with urine. Uterus size of new-born infant's head, soft, flabby, filling pelvic cavity, and containing a large four-ounce coagulum, fastened to place of placental attachment; large blood clots in vagina, and great congestion, but no other sign of inflammation. Mammæ filled with milk.

In left knee-joint about sixteen grammes of clear serum, with a little fibrin, showing under the microscope fibrin and dividing white blood cells; in right knee-joint a small quantity of synovial fluid, and also in both elbows.

*Remarks:* This patient was eight months pregnant, and had an attack of inflammatory rheumatism, which caused her to abort. Only the left knee-joint was affected. Her death was from asphyxia, to which the spots of Tardieu bear witness. The death was not sudden, but was unexpected to the attendant. The literature upon death from air in the veins is surprisingly small, and with regard to the morbid anatomy in such cases, I find nothing. Dö the air bubbles act as emboli? Were they the cause of the two infarcts in the right lung? I experimented with water mixed with air, forced gently through glass tubes, which I drew out into the finest possible capillary tubes. The air, of course, passed through readily, the bubbles elongating and minutely dividing. Still it may be vastly different in the microscopic capillaries of the lungs. The body was frozen, therefore no decomposition had taken place.

### NO. 22—INTERNAL ABDOMINAL HERNIA.

Insane Asylum, Feb. 25—H. C., female, æt. 60; dead nineteen hours. Great emaciation of the body and anæmia.

Brain not examined.

*Chest:* Pleural surfaces normal; emphysema of lungs from senile atrophy; in left pleural cavity small quantity of bloody serum. Slight thickening of some valves of heart; atheromatous degeneration of aorta; fatty degeneration of myocardium; no clots and no blood in heart or large vessels.

*Abdomen:* Spleen normal. Fatty degeneration and senile atrophy of liver. Granular atrophy of both kidneys. Aorta at bifurcation of iliacs solid with lime from atheromatous process. Bladder thin-walled and distended with urine. Ovaries and uterus atrophied. Slight chronic gastritis. From omentum majus going down to mesentery of sigmoid flexture was a cord-like piece of tissue, about four mm. thick, congested, which formed a circular aperture about seven cm. in diameter, and through which about half of the loops of the ileum projected, thus forming an internal hernia.

*Diagnosis:* Emphysema; granular atrophy of kidneys; anæmia.

*Remarks:* The chief interest is, of course, the internal abdominal hernia. There were no signs of old adhesive inflammation, and the malformation was probably congenital. The narrow cord itself, however, had grown hard from constant and chronic irritation. In the history of the case was nothing to show that this had ever caused any trouble. The aperture was too large to compress in any way the canal of the ileum.

### NO. 23—ALVEOLAR SARCOMA OF SEROUS MEMBRANES IN BOY OF TWELVE YEARS.

Patient of Dr. Tobie, March 13—Kearns, æt. 12, male, Irish-American; dead ten hours. Body very anæmic and emaciated; arms thin; legs, scrotum, penis and abdomen œdematous to enormous degree; veins on abdomen, neck and forehead, extremely enlarged.

Brain not examined.

*Chest:* In each pleural cavity some 300 ccm. of sanguinous fluid; upon the pleuræ, in different portions, numerous millet-seed growths, some as large as peas, and extending into lung tissue, of white, medullary nature; on right side, adhesions at apex; no tumors in lungs themselves; lungs congested and œdematous; heart and valves normal; a few white agonia clots; in pericardium some 150 ccm. of clear serum; pericardium itself normal.

*Abdomen:* Contained sixteen litres or more of sanguino-purulent fluid; peritoneum inflamed; everywhere covered with thousands of growths, from size of a pea to ten and even fifteen cm. in diameter, of soft medullary consistence; the diaphragm on abdominal surface one mass of this tumor one and one-half cm. in thickness. Liver, gorged with blood, contained a few of these nodules in its capsule, extending into the liver substance itself, with undefined edges, as those of pleuræ into lungs. The omentum majus was thickly covered with these. The bladder had become one mass of the growth fifteen cm. in diameter, leading to dilatation of ureters and hydronephrosis. The mesentery everywhere covered with large and small tumors, involving the loops of the ileum in their masses. Spleen normal. Stomach normal. Gall-bladder had become a mass of this white tumor and was not to be found.

*Diagnosis:* With the microscope there was no difficulty in recognizing the tumor as alveolar sarcoma.

*Remarks:* Such extensive malignant disease of pleuræ and peritoneum in a child is remarkably rare. Six months before his death the patient had scarlet fever. Previous to this he was perfectly healthy. Four months ago the tumor began to develop. There are one or two cases on record where sarcoma has rapidly developed as a sequel to scarlatina.

NO. 26—A PATHOLOGICAL MUSEUM. HERMAPHRODITISMUS SPURIUS.

General Hospital, March 21—Henry S., sailor, æt. 50; body well nourished; lower extremities œdematous.

Brain not examined.

*Chest:* In left pleural cavity two litres of serum; partial atalectasis of left lung; no adhesions; in right cavity three litres of fibro-purulent serum; right pleura everywhere covered with pus and fibrin; in right lung atalectasis complete, combined with caseous pneumonia. Heart hypertrophied on both sides; stenosis of mitral valve, hardly passing one finger; one wing of mitral valve contained a deposit of lime five mm. thick and two by one cm. in length and breadth, over this being a large atheromatous ulcer with vegetations; left and right hearts filled with soft post mortem clots; tricuspid and pulmonary valves normal; thickening and stenosis of aortic, each lunette containing calcareous deposit; beginning atheromatous process in aortic arch; weight of heart 496 grammes or fifteen and one-half ounces.

*Abdomen:* Spleen atrophied, weighing ninety-six grammes. Liver somewhat fatty and enlarged, weighing three and three-fourths pounds. In right kidney scars of old cysts and granular atrophy; in left kidney one colloid cyst, four scars, and granular atrophy. Bladder very small, with hypertrophied walls one and one-fourth cm. thick, and cavity not larger than a hazel nut, containing some pus. Contraction of ascending colon (cœcum being of normal size) of transverse and descending colon and rectum to size of little finger, from large old ulcers five by seven cm. in dimensions; just above chief stricture in ascending colon an irritative inflammation of mucous membrane; ileum showed no signs of previous ulceration.

Penis not more than eight cm. in length; had no prepuce, and only a groove on under side for a urethra; urethral opening at the perineo-scrotal junction about three cm. in front of anus; this was large enough to admit of passing one finger directly into bladder. Both testes had but just reached a little way into the inguinal opening, and lay with their epididymes upon the pubic bone; testes no larger than beans, somewhat surpassed in size by the epididymes; on the membranes about both were several pellucid colloid cysts.

*Diagnosis:* Caseous pneumonia giving rise to pleurisy, and empyema immediate cause of death; mitral and aortic stenosis leading to œdema of lower extremities, and cardiac hypertrophy; intestinal obstruction as a result of former ulceration (kind unknown). Finally, non-descent of testes and hypospadia.

*Remarks:* The penis was so small as to pass for an enlarged clitoris, and the urethal opening might easily have been mistaken by an ignorant person for a vagina. There was no scrotum, and the small testes on the pubic bone caused the pubic region to appear like a mons veneris. It was in fact a case of hermaphroditismus spurius.

NO. 31—DEATH AFTER RESUSCITATION FROM DROWNING.

General Hospital, April 18—Alfonso Vienocolo, laborer, æt. 22; dead seventeen hours; body well built, small, well nourished; rigor mortis still present.

Brain not examined.

*Chest:* Acute double pneumonia and bronchitis; stage of red hepatization; slight fatty degeneration of heart.

*Abdomen:* Spleen normal; liver, in parts, fatty, greatly congested and minute subcapsular hemorrhages in great number; kidneys soft and fatty; bladder greatly distended with urine; venous engorgement everywhere.

*Diagnosis:* Acute double pneumonia, caused by water inhalation.

*Remarks:* The man was drowned, immediately resuscitated, and died two days after the accident.

(*To be continued in next number of Journal.*)

